# Impact of beta-lactam allergy labels on bone marrow transplant patients

**DOI:** 10.1017/ash.2025.172

**Published:** 2025-06-16

**Authors:** Benjamin M. Haxby, Kendall J. Tucker, Caitlin M. McCracken, YoungYoon Ham, Jon P. Furuno, Jessina C. McGregor

**Affiliations:** 1 Department of BioHealth Sciences, College of Science, Oregon State University, Corvallis, OR, USA; 2 Department of Pharmacy Practice, College of Pharmacy, Oregon State University, Portland, OR, USA; 3 Department of Pharmacy Services, Oregon Health & Science University, Portland, OR, USA

## Abstract

**Background:**

Approximately 95% of patients with a beta-lactam allergy noted in their medical record are not truly allergic when tested. These patients may unnecessarily avoid first-line antibiotics, resulting in increased treatment failure, higher costs, and antibiotic resistance. Bone marrow transplant (BMT) patients may be at higher risk for these adverse outcomes due to weakened immune systems and high risk for severe infections. Our objective was to evaluate beta-lactam allergy labels and their influence on BMT patient outcomes.

**Methods:**

We conducted a retrospective cohort study of adult inpatients undergoing BMT during April 2018-March 2020. Eligibility for penicillin allergy testing/de-labeling was evaluated. Multivariable logistic regression was performed to measure independent effects of beta-lactam allergy labels on 100-day outcomes: mortality, ICU admission, rehospitalization, and intravenous antibiotic use.

**Results:**

Among 358 BMT patients, 75 (21%) had a beta-lactam allergy label at baseline. Mortality was higher in patients with an allergy label (14.7% vs 7.8%, *P* = 0.067). In multivariable analysis, patients with allergy labels were not at a significantly greater risk of mortality (OR = 1.60; 95% CI = 0.68 – 3.78) but were significantly more likely to receive carbapenems (OR = 6.27; 95% CI = 2.81–13.98). All patients with penicillin-class allergy labels were eligible for allergy testing/de-labeling.

**Conclusion:**

We did not observe a significant increased risk of mortality in BMT patients with beta-lactam allergy labels; however, increased carbapenem use was observed. Penicillin allergy de-labeling programs may help optimize antibiotic prescribing in BMT patients. Larger studies are needed to quantify the impact of beta-lactam allergy labels on BMT patient outcomes.

## Introduction

Beta-lactam antibiotic allergies are reported in up to 20% of hospitalized patients of which penicillin and cephalosporin allergies make up approximately 10%–15% and 1%–2% of these recorded allergies, respectively.^1–4^ However, allergy documentation in medical records has been widely demonstrated to inaccurately document allergies. Only about 25%–45% of patients with a recorded penicillin allergy in the medical record, *ie*, a penicillin allergy label, report true immune-mediated allergic reactions.^4–6^ Also, since IgE-mediated reactions to penicillin have been shown to fade over time with 80% of patients losing hypersensitivity after 10 years, older documented allergies may no longer be clinically relevant.^7,8^ Facilities that have implemented penicillin allergy testing programs have identified roughly 95% of patients with a recorded penicillin allergy can safely receive penicillin and other beta-lactam antibiotics, such as cephalosporins.^3,5,6,9–11^ The high proportion of misreported allergy labels paired with waning sensitivity of IgE-mediated reactions results in many patients having penicillin allergy labels that improperly reflect their true allergy status.

Beta-lactam antibiotics such as penicillins and cephalosporins are the preferred treatment for many bacterial infections because they are safer, more effective, and more specific when compared to alternative antibiotics.^9,12–14^ The presence of a beta-lactam allergy label can influence provider selection of antimicrobial treatment. This results in decreased usage of penicillins and cephalosporins and higher usage of second-line antibiotics such as vancomycin, clindamycin, fluoroquinolone, and carbapenem.^2–4,15^ Beta-lactam allergy labels have also been associated with worse patient outcomes, higher healthcare costs, and higher infection rates from multi-drug resistant organisms.^2,4,15–20^ One study of hospitalized patients with hematologic malignancies found patients with a beta-lactam allergy label were hospitalized on average 4 days longer than patients without a beta-lactam allergy label and were 1.6 times more likely to die.^2^ The negative effects observed in this population could be exaggerated for patients who require a bone marrow transplant procedure (BMT).

BMT is performed over 20,000 times per year in the United States.^21^ Following transplant, patients suffer from severe neutropenia and are at a higher risk of serious infection. Within 100 days post-BMT, infections are responsible for an estimated 20% of the deaths.^21^ A weakened immune system coupled with extended hospital exposure means BMT patients could suffer worse consequences from beta-lactam allergy labels and the associated lack of preferential antibiotic therapy that studies in other populations have shown.^2,4,15,18,19^

While other studies have detailed the risks associated with allergy labels in general inpatient populations, this study expands on how beta-lactam allergy labels impact patients undergoing BMT. Our objective was to quantify the association between beta-lactam allergy labels and clinical outcomes in BMT patients. Additionally, we quantified the potential for allergy de-labeling in this population by (1) evaluating documentation of individual allergy labels to confirm presence of a true allergy and (2) identify potential for oral challenge or skin testing.

## Methods

### Study design and inclusion/exclusion criteria

We conducted a retrospective cohort study of inpatients that received a BMT procedure between April 2018 and March 2020. The study site is a 576-bed quaternary care, academic medical center. Patients under the age of 18 at the time of transplant were excluded. During the study period, an established hospital antimicrobial stewardship program was in place; a febrile neutropenia protocol, collaboratively developed with the BMT physician teams, called for meropenem in patients with severe Ig-E mediated reactions (anaphylaxis, angioedema) and cefepime in patients with less severe reactions (eg rash, hives). Approval for the study was obtained from the local Institutional Review Board.

### Data collection

Eligible study subjects were identified using a research repository of continuously refreshed longitudinal data of inpatient and outpatient electronic health record (EHR) data. Repository data included patient demographics, ICD-10 diagnosis codes, antibiotic allergy labels, pharmacy data, and transplant procedure data. In addition, we manually reviewed patients’ EHRs and collected data on patient outcomes and allergy labels which were entered into a REDCap data collection form.^22,23^ Allergy label data included the specific drug allergy, date of reaction, reaction type, sufficient characterization, and eligibility for penicillin allergy testing per institutional protocol (appendix 1).^11^

### Variable definitions

Our primary exposure of interest was the presence of a beta-lactam allergy label in the patient’s EHR prior to the BMT. Allergy label reaction type was classified from the documented reaction as IgE-mediated, non-IgE-mediated, undifferentiated rash, adverse reaction, or unknown based on institutional protocol (appendix 1).^11^ IgE- and non-IgE-mediated reactions were further classified as mild or severe based on reaction descriptions and classifications within the allergy label. The adverse reactions category primarily included common antibiotic side effects such as diarrhea, nausea, and vomiting. Allergies were classified as sufficiently characterized if both the reaction and date of reaction were described. Specific notes for when the allergy occurred were required to differentiate between the reaction date and when the allergy label was entered in the medical record. Eligibility for penicillin allergy testing was determined using an institutional protocol (appendix 1) and was based on the reaction type and when the reaction occurred.^11^ Eligibility for cephalosporin testing was not evaluated. Two infectious disease pharmacists assisted with evaluating allergy type, severity, and eligibility for penicillin testing.

Our outcomes of interest were mortality, readmission, hospitalization days following transplant, intensive care unit (ICU) admission, *C. difficile* infection, development of fever, graft vs host disease, and antibiotic usage. All outcomes were assessed using healthcare-system wide data within the 100-day period following the BMT procedure except mortality which was additionally assessed within 30 days of BMT. ICU admission was only recorded for patients after receipt of their first antibiotic. Antimicrobial treatment data was recorded only for intravenous antibiotics to isolate treatment for suspected infections rather than prophylaxis. Median days of total and specific IV antibiotic use was calculated from patients that received at least one day of the measured antibiotic. Subsequent readmissions and health care encounters that occurred outside of the healthcare system following transplant could not be accounted for.

We also collected data on patient demographics and comorbidities present at time of admission for transplant. Transplant type was identified as autologous or allogeneic. Transplant indication was categorized as: acute lymphoid leukemia, acute myeloid leukemia, non-Hodgkin’s lymphoma, Hodgkin’s lymphoma, multiple myeloma, and other. Rare forms of cancer and nonmalignant diseases were included in the “other” category. Patients with myelodysplastic syndromes were included in the acute myeloid leukemia group due to similarities between the conditions.

### Statistical analysis

BMT patients with and without a beta-lactam allergy label present in their EHR at the time of transplant were initially described using frequencies for categorical data and medians and interquartile ranges for continuous variables. Continuous data were compared using Wilcoxon-rank sum tests, while 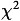 or Fischer exact tests were used to compare categorical data. Statistical significance was defined as *P* < 0.05.

Multivariable logistic regression models were used to evaluate the independent effect of beta-lactam allergy labels on clinical outcomes. Separate models were constructed for 100-day mortality, ICU admission, readmission, and carbapenem use, while adjusting for confounding variables. A stepwise model building approach was utilized. Confounders were retained in the model if they individually resulted in a greater than 10% change in the effect of allergy labels on the outcome of interest. All variables associated with the outcome of interest at the p < 0.2 level in bivariable analysis were considered for entry into the multivariable model. Reference groups varied between models with the most protective variable level being chosen as the reference. Variables remained in the model if they stayed below a significance level of p < 0.05 or were identified as a confounder. Allergy label was retained in all models. All statistical analysis was conducted using SAS v9.4 (Cary, NC).

## Results

### Demographics and comorbidities

In total, 358 patients met our inclusion criteria and were included in this study (Table 1). Overall, 75 (20.9%) patients had at least one beta-lactam allergy label prior to transplant; 59 (16.5%) patients had a penicillin allergy label, and 21 (5.9%) patients had a cephalosporin allergy label. Note that 5 (1.4%) of these patients had reported an allergy to both penicillin and cephalosporin. Patients with and without allergy labels were comparable for age, body mass index, obesity, heart disease, diabetes, hematologic malignancy, and multiple cancer types. Patients with an allergy label had higher prevalence of chronic kidney disease (21.3% vs 14.5%), allogenic transplants (61.3% vs 49.5%), and acute myeloid leukemia (45.3% vs 31.1%) when compared to patients without an allergy label. Conversely, the non-allergy label group had more male patients (56.9% vs 45.3%), autologous transplants (50.5% vs 38.7%), and multiple myeloma (32.9% vs 17.3%).

**Table 1.** Characteristics of hospitalized patients receiving bone marrow transplant (BMT) stratified by presence of allergy label on admission

	No beta-lactam allergy label on admission (N= 283)	Beta-lactam allergy label on admission (N = 75)	P-value
Age (median, IQR)	60 (46–67)	61 (46–68)	0.982
Sex (n, %)			
Male	161 (56.9)	34 (45.3)	0.074
Female	122 (43.1)	41 (54.7)	0.074
Race (n, %)			
White	251 (88.7)	66 (88.0)	0.867
Other	32 (11.3)	9 (12.0)	0.867
BMT encounter length of stay (median, IQR)	19 (15–24)	21 (16–24)	0.219
Body mass index (median, IQR)	28 (25–33)	27 (23–31)	0.139
Obesity^a^ (n, %)	98 (34.6)	24 (32.0)	0.669
Chronic kidney disease (n, %)	41 (14.5)	16 (21.3)	0.150
Diabetes mellitus (n, %)	34 (12.0)	10 (13.3)	0.757
Chronic liver disease (n, %)	12 (4.2)	2 (2.7)	0.532
Heart disease (n, %)	120 (42.4)	34 (45.3)	0.649
Hematologic malignancy (n, %)	260 (91.9)	67 (89.3)	0.487
Transplant type (n, %)			
Autologous	143 (50.5)	29 (38.7)	0.068
Allogenic	140 (49.5)	46 (61.3)	0.068
Transplant indication (n, %)			
Acute lymphoid leukemia	26 (9.2)	6 (8.0)	>0.99
Acute myeloid leukemia	88 (31.1)	35 (45.3)	0.012
Non-Hodgkin’s lymphoma	34 (12.0)	10 (13.3)	0.757
Hodgkin’s lymphoma	18 (6.4)	4 (5.3)	>0.99
Multiple myeloma	93 (32.9)	13 (17.3)	0.001
Other	24 (8.5)	7 (9.3)	0.736
New allergy documented within 100 days post-transplant (n, %)	11 (3.9)	3 (4.0)	>0.99

a *Obesity defined as a body mass index* 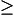 *30.*

### Characteristics of allergy labels

**Figure 1.**
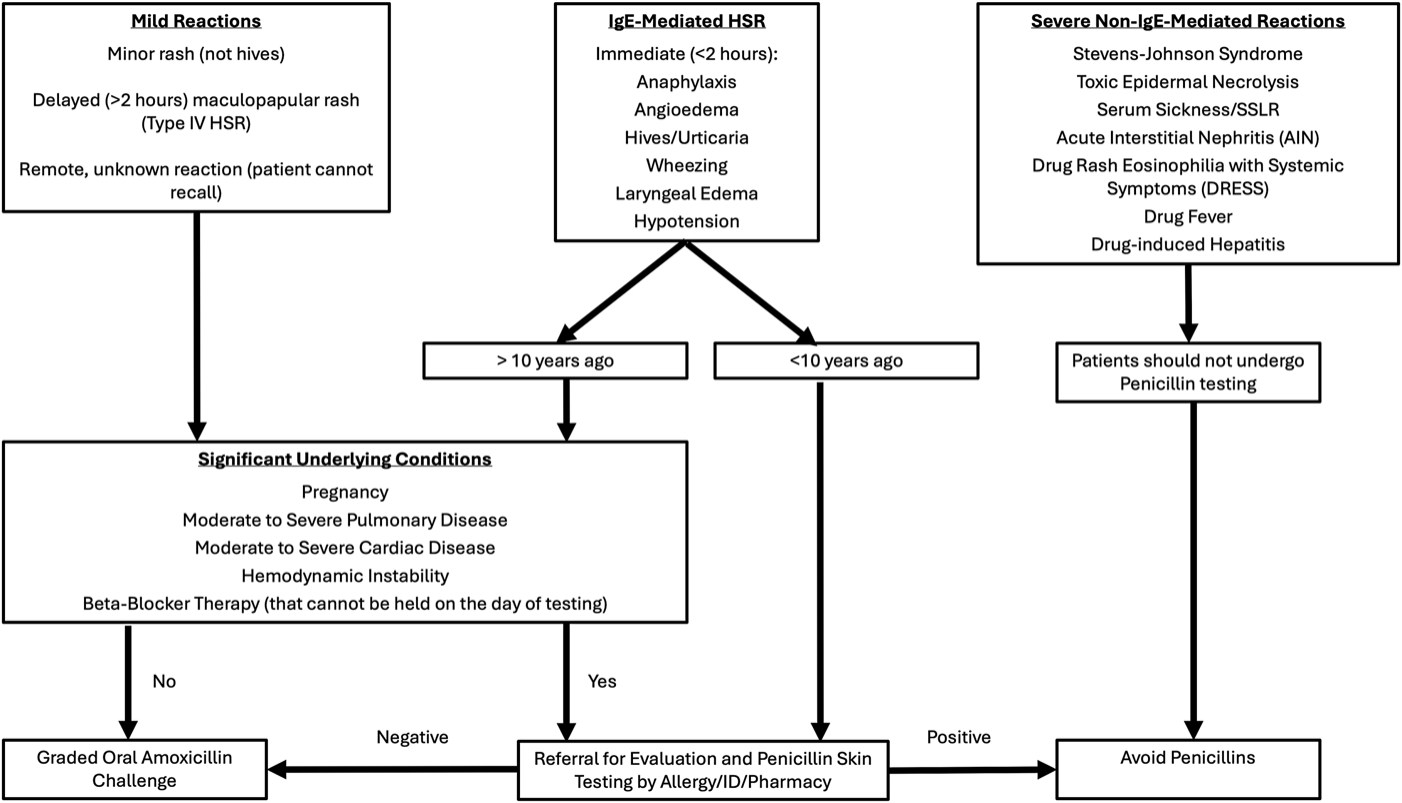
Institutional protocol for determining patient eligibility for Penicillin allergy testing.

There were 92 individual beta-lactam allergy labels recorded from the 75 study patients with a beta-lactam allergy label prior to transplant. There were 66/92 (71.7%) penicillin allergy labels and 26/92 (28.3%) cephalosporin allergy labels (Table 2). Non-IgE-mediated allergic reactions were not documented in any of the 92 allergy labels. IgE-mediated allergic reactions were documented in 19/66 (28.8%) and 5/26 (19.2%) of penicillin and cephalosporin allergy labels. Of these IgE-mediated reactions, 9/66 (13.6%) penicillin labels and 3/26 (11.5%) cephalosporin labels were documented as severe. Undifferentiated rash was more common in the cephalosporin allergy group with 20/26 (76.9%) labels reporting this reaction compared to 38/66 (57.6%) in penicillin labels. Both penicillin and cephalosporin allergy groups shared a high proportion of insufficiently characterized allergy labels at 64/66 (97%) and 26/26 (100%), respectively. An unspecified date of reaction (93.9%, 100%) was a much more common reason for this than the reaction not being described (7.6%, 3.8%). Based on institutional protocol (Figure 1), 66/66 (100%) of the penicillin allergy labels were eligible to be tested for accuracy and delabeled if no true allergy is present.

**Table 2.** Characteristics of individual patient allergy labels prior to bone marrow transplant stratified by drug class

	Penicillin (N = 66)	Cephalosporin (N = 26)
Reaction type (n, %)		
Mild non-IgE	0	0
Severe non-IgE	0	0
Mild IgE	10 (15.2)	2 (7.7)
Severe IgE	9 (13.6)	3 (11.5)
Adverse reaction	8 (12.1)	3 (11.5)
Undifferentiated rash	38 (57.6)	20 (76.9)
Unknown	3 (4.5)	0
Other	1 (1.5)	0
Reaction type and comment mismatch (n, %)	1 (1.5)	0
Label not sufficiently characterized (n, %)	64 (97.0)	26 (100)
Date of reaction not specified	62 (93.9)	26 (100)
Reaction not described	5 (7.6)	1 (3.8)
Prior to transplant patient received antibiotic of same class (n, %)	4 (6.1)	3 (11.5)
Potential for penicillin allergy testing (n, %)	66 (100)	NA

Table 2 *is measured at the allergy level. Some patients have more than 1 allergy label in their medical chart.*

### Antibiotic usage

Antibiotic use varied significantly between the allergy label and non-allergy label groups (Table 3). Patients without a beta-lactam allergy label were more likely to receive penicillin (28.6% vs 13.3%, *P* = 0.007) and cephalosporin (74.2% vs 56%, *P* = 0.002) compared to patients with a beta-lactam allergy label. Conversely, beta-lactam allergy label patients were more likely to receive carbapenems (25.3% vs 5.7%, *P* < .001), metronidazole (25.3% vs 14.5%, p = 0.021), clindamycin (4% vs 0%, *P* < 0.01), and fluoroquinolone (24% vs 18.7%, *P* = 0.31). This group also had higher median days of fluoroquinolone therapy (5 vs 3, *P* = 0.137) and resistant gram-positive therapy (7 vs 4, *P* = 0.153), although these results were not statistically significant. No difference was observed between the two groups for total days of antibiotic therapy or median number of antibiotic classes used.

**Table 3.** IV antibiotic usage 100 days post-transplant stratified by presence of allergy label prior to transplant

	No beta-lactam allergy label on admission (N = 283)	Beta-lactam allergy label on admission (N = 75)	P-value
Any intravenous antibiotic use (n, %)	222 (78.4)	58 (77.3)	0.836
Total days of antibiotic therapy (median, IQR)	9 (3–17)	9 (3–19)	0.508
Total antibiotic classes (median, IQR)	2 (1–3)	2 (1–3)	0.546
Penicillin use (n, %)	81 (28.6)	10 (13.3)	0.007
Days of penicillin (median, IQR)	5 (3–8)	9 (2–14)	0.562
Cephalosporin use (n, %)	210 (74.2)	42 (56.0)	0.002
Days of cephalosporins (median, IQR)	7 (4–10)	7 (5–15)	0.229
Carbapenem use (n, %)	16 (5.7)	19 (25.3)	<0.001
Days of carbapenem (median, IQR)	5 (3–11)	6 (4–10)	0.816
Aminoglycoside use (n, %)	0	0	–
Aztreonam use (n, %)	3 (1.1)	1 (1.3)	–
Ceftolozane—Tazobactam use (n, %)	2 (0.7)	2 (2.7)	–
Fluoroquinolone use (n, %)	53 (18.7)	18 (24.0)	0.309
Days of fluoroquinolone (median, IQR)	3 (2–6)	5 (3–7)	0.137
Clindamycin use (n, %)	0	3 (4.0)	0.008
Resistant-Gram-Positive Use (n, %)	71 (25.1)	19 (25.3)	0.965
Days of resistant Gram-positives (median, IQR)	4 (2–7)	7 (2–13)	0.153
metronidazole Use (n, %)	41 (14.5)	19 (25.3)	0.021
Days of metronidazole (median, IQR)	6 (4–8)	5 (4–8)	0.767
Macrolide Use (n, %)	3 (1.1)	1 (1.3)	

The results of the multivariable logistic regression model identifying independent predictors of carbapenem usage is show in Table 4; antibiotic allergy label and gram-negative blood culture were identified as independent risk factors for receiving carbapenem antibiotics. Patients with an allergy label were 6.3 times more likely to receive a carbapenem (odds ratio [OR] = 6.27; 95% confidence interval [CI] = 2.81 – 13.98; *P* < 0.0001), while patients who had a gram-negative blood culture were 13.8 times as likely (OR = 13.79; 95% CI = 3.96 – 48.08; *P* = 0.019). Gram-negative blood culture and transplant indication were identified as confounders of allergy labels impact on carbapenem antibiotics. Non-Hodgkin’s lymphoma served as the reference group against other transplant indications.

**Table 4.** Independent predictors of carbapenem treatment in logistic regression analysis

Predictor of receiving carbapenem	OR	95% CI	p-value
Allergy label	6.27	2.81–13.98	<0.001
Gram-negative blood culture*	13.79	3.96–48.08	0.019
Transplant indication*			
Acute lymphocytic leukemia	6.34	1.05–38.25	0.070
Acute myeloid leukemia	4.40	0.90–21.45	0.118
Hodgkin’s lymphoma	1.69	0.13–21.62	0.654
Multiple myeloma	1.26	0.20–7.75	0.178
Other	4.45	0.69–28.65	0.297
Non-Hodgkin’s lymphoma	REF	–	–

*Independent predictors of receiving carbapenem antibiotics within 100 days following bone marrow transplant.*

* *Denotes confounding variables on the primary exposure variable allergy label.*

### Treatment-related outcomes

Both patient groups were comparable for several treatment related outcomes, and no outcomes yielded statistically significant differences below the *P* = 0.05 level (Table 5). The greatest difference between the two groups was for 100-day mortality where death occurred at a higher rate in the beta-lactam allergy label group (14.7% vs 7.8%, *P* = 0.067). The beta-lactam allergy label group also showed higher rates for ICU admission (14.7% vs 11%, *P* = 0.374), graft vs host disease (16% vs 13.1, *P* = 0.512), and readmission (25.3% vs 22.3%, *P* = 0.574). Medians for days with fever (3 vs 2, *P* = 0.472) and days with low absolute neutrophil count (15 vs 13, *P* = 0.451) were also higher in the beta-lactam allergy label group. The non-BL allergy group had a higher rate of *C. difficile* infection (5.7% vs 2.7%, *P* = 0.385). Little to no difference was observed between the groups for median days hospitalized after transplant, 30-day mortality, new acute kidney injury, and positive blood culture.

**Table 5.** Patient outcomes 100 days post-transplant stratified by presence of allergy label prior to transplant

	No beta-lactam allergy label on admission (N = 283)	Beta-lactam allergy label on admission (N = 75)	p-value
Readmission (n, %)	63 (22.3)	19 (25.3)	0.574
Died during index encounter (n, %)	9 (3.2)	3 (4.0)	0.721
30-day mortality (n, %)	9 (3.2)	4 (5.3)	0.484
100-day mortality (n, %)	22 (7.8)	11 (14.7)	0.067
Median days hospitalized post-transplant (n, %)	16 (13–22)	16 (13–25)	0.646
Fever occurrence (n, %)	186 (65.7)	47 (62.7)	0.621
Days with fever (median, IQR)	2 (1–4)	3 (1–4)	0.472
^a^Low absolute neutrophil count occurrence (n, %)	283 (100)	75 (100)	
^a^Days of low absolute neutrophil count (median, IQR)	13 (9–22)	15 (9–23)	0.451
Transferred to ICU after first Abx (n, %)	31 (11.0)	11 (14.7)	0.374
New acute kidney injury (n, %)	49 (17.3)	12 (16.0)	0.788
*Clostridium difficile* infection (n, %)	16 (5.7)	2 (2.7)	0.385
Graft Vs Host disease (n, %)	37 (13.1)	12 (16.0)	0.512
Microbiology (n, %)			
Positive blood culture (n, %)	45 (15.9)	13 (17.3)	0.765
Gram positive org (n, %)	29 (10.3)	12 (16.0)	0.164
Gram negative org (n, %)	13 (4.6)	3 (4.0)	>0.99
Grew anaerobe (n, %)	9 (3.2)	2 (2.7)	>0.99
Grew fungus (n, %)	3 (1.1)	1 (1.3)	>0.99

All variables are measured in the 100-day period following bone marrow transplantunless otherwise specified.

a Low absolute neutrophil count defined as < 500.

Independent Predictors of 100-day mortality are included in Table 6. Mortality within 100 days of transplant was 1.6 times more likely (OR = 1.60; 95% CI = 0.68 – 3.78; *P* = 0.28) in patients with a beta-lactam allergy label, although this relationship was not statistically significant. Chronic kidney disease was a strong predictor of 100-day mortality (OR = 8.78; 95% CI = 3.74 – 20.59; *P* < 0.0001). Chronic kidney disease, transplant indication, and transplant type were identified as confounders of allergy label’s effect on 100-day mortality. Autologous transplant was the most protective transplant type, and multiple myeloma was the most protective transplant indication for 100-day mortality. Multivariable analysis did not identify the presence of a beta-lactam allergy label to be an independent predictor of readmission (eTable 1) or ICU admission (eTable 2) within 100 days of transplant.

**Table 6.** Multivariable regression for 100-day mortality

Predictor of 100-day mortality	OR	95% CI	p-value
Allergy label	1.60	0.68–3.78	0.280
Chronic kidney disease*	8.78	3.74–20.59	<0.001
Transplant type*			
Allogeneic transplant	1.30	0.25–6.88	0.760
Autologous transplant	REF	–	–
Transplant indication*			
Acute lymphocytic leukemia	22.52	1.35–376.8	0.946
Acute myeloid leukemia	17.44	1.22–249.3	0.951
Hodgkin’s lymphoma	< 0.001	<0.001–>999.9	0.962
Non-Hodgkin’s lymphoma	11.19	1.18–106.6	0.960
Other	24.70	1.81–337.7	0.944
Multiple myeloma	REF	–	–

*Independent predictors of mortality within 100 days following bone marrow transplant.*

* *Denotes confounding variables on the primary exposure variable allergy label.*

## Discussion

In this study, we did not observe a significant association between beta-lactam allergy labels and increased risk of poor patient outcomes among BMT patients. Although not statistically significant, mortality within 100 days of transplant occurred in 15% of the allergy label group which was nearly double the rate seen in patients without beta-lactam allergy labels (*P* = 0.067). Larger studies in other patient populations have previously demonstrated the negative impacts of antimicrobial allergy labels on patient outcomes. While our results were more inconsistent for outcomes such as length of stay, ICU admission, readmission, and *C. difficile* infection when compared to prior research, this may be due to either an insufficient sample size or broader inclusion criteria.^2,4,15,18,15–18^

Intravenous antibiotic utilization varied significantly between patients with and without a beta-lactam allergy label in their medical record. After adjusting for transplant indication and gram-negative blood culture, patients with a beta-lactam allergy label were 6.3 times more likely to receive carbapenem antibiotics than patients without an allergy label. This marks a significant increase over what has been reported for non-BMT populations.^3,15,24^ Blumenthal et al. conducted a study of 11,000 general inpatients at over 100 hospitals and observed that patients with a penicillin allergy label were only 1.83 times more likely to receive carbapenem antibiotics.^3^ The prevalence of beta-lactam allergy labels prior to transplant in our cohort was 21%, with penicillin and cephalosporin allergy labels present in 16.5% and 5.6% of patients, respectively. These values are higher than previously reported in general inpatient populations.^1,4,18,25^ All patients with a documented allergy to penicillin in our study were eligible for either a graded oral amoxicillin challenge or penicillin skin test. Prior research suggests that as many as 95% of patients with a penicillin allergy label in their EHR test negative and could be de-labeled when subject to a penicillin skin or oral challenge.^3,5,6,9–11^ The higher frequency of penicillin allergy labels, coupled with the significantly increased likelihood of carbapenem use among those with the label, highlights the importance of de-labeling programs specifically within BMT patient populations.

In addition to the potential antimicrobial stewardship implications of de-labeling, the cost-savings benefits associated with the removal of allergy labels have also been previously demonstrated.^5,9,26^ A UK study by Li et al. identified that at least 40% of patients could have penicillin allergy labels removed from patient medical records following structured interviews, and these patients had antibiotic expenditures that were 1.82 to 2.58-fold higher than if they had used first-line agents.^27^ The authors calculate that de-labeling could reduce antibiotic costs by £5,851.18 to £14,471.93 for each patient.^27^ If similar savings are observed in the United States, this represents meaningful savings for hospitals compared to the one-time cost of $145-$220 for penicillin allergy testing.^28^

Several limitations should be considered when interpreting the validity of our study. First, the retrospective nature and lack of sufficient allergy history in our study made us unable to confirm the validity of patient allergies or know which allergy labels influenced clinical decision making. Although present, some providers may choose to disregard patient allergy labels if the risk of alternative antibiotic therapy poses a greater threat to the patient than a potential allergic reaction. Given this, some patients in the study were likely treated as if they did not have a beta-lactam allergy even though they were retained in the allergy label group. Second, the data in our study was confined to the healthcare system. Thus, we could fully capture historical antibiotic use, and any readmissions or subsequent healthcare utilization that occurred outside of our healthcare system in the 100-day window following the initial transplant encounter would not have been captured in our analysis and could result in our outcomes being under-reported. Finally, we classified allergy label status immediately prior to transplant. Our analysis did not allow for time-varying classification of exposure status, so a small number of patients who developed a new allergic reaction, and hence a new allergy label during the follow-up period, were retained in the no allergy group (Table 1). Due to our small sample size, we chose not to exclude these patients from the study, but their allergic reactions and subsequent changes in antibiotic therapy may have impacted our outcomes.

In conclusion, our study aimed to evaluate the impact of beta-lactam allergy labels on BMT patient outcomes. Additionally, we examined individual beta-lactam allergy labels to understand how they are characterized and evaluate patient potential for penicillin allergy testing. We observed a non-significant trend towards increased 100-day mortality rates among patients with a beta-lactam allergy label, potentially attributable to sub-optimal antimicrobial treatment following BMT. Patients with a beta-lactam allergy label were less likely to receive penicillin and cephalosporin class antibiotics while being more likely to receive alternative antibiotics such as carbapenems and clindamycin. All patients with a penicillin allergy label in this study were eligible for allergy testing. To decrease the number of patients that report beta-lactam allergies in EHRs, healthcare systems should focus on improved allergy documentation and implementation of penicillin allergy de-labeling programs. Past studies have shown that penicillin allergy testing can improve patient outcomes, optimize antibiotic utilization, and reduce healthcare costs. This could be especially true for vulnerable populations, such as those undergoing BMT, that are at a high risk of infection and would benefit from having as many antimicrobial treatment options as possible.

## Supporting information

10.1017/ash.2025.172.sm001 Haxby et al. supplementary material Haxby et al. supplementary material

## Supplementary material

The supplementary material for this article can be found at https://doi.org/10.1017/ash.2025.172.

## References

[1] Lee CE , Zembower TR , Fotis MA , et al. The incidence of antimicrobial allergies in hospitalized patients: implications regarding prescribing patterns and emerging bacterial resistance. Arch Intern Med 2000;160:2819–2822. 11025792 10.1001/archinte.160.18.2819

[2] Huang KG , Cluzet V , Hamilton K , Fadugba O . The impact of reported beta-lactam allergy in hospitalized patients with hematologic malignancies requiring antibiotics. Clin Infect Dis 2018;67:27–33. 29346543 10.1093/cid/ciy037

[3] Blumenthal KG , Kuper K , Schulz LT , et al. Association between Penicillin allergy documentation and antibiotic use. JAMA Intern Med 2020;180:1120–1122. 32597920 10.1001/jamainternmed.2020.2227 PMC7325068

[4] MacFadden DR , LaDelfa A , Leen J , et al. Impact of reported beta-lactam allergy on inpatient outcomes: a multicenter prospective cohort study. Clin Infect Dis 2016;63:904–910. 27402820 10.1093/cid/ciw462

[5] Rimawi RH , Cook PP , Gooch M , et al. The impact of penicillin skin testing on clinical practice and antimicrobial stewardship. J Hosp Med 2013;8:341–345. 23553999 10.1002/jhm.2036

[6] Blumenthal KG , Shenoy ES , Varughese CA , Hurwitz S , Hooper DC , Banerji A . Impact of a clinical guideline for prescribing antibiotics to inpatients reporting penicillin or cephalosporin allergy. Ann Allergy Asthma Immunol 2015;115:294–300 e292. 26070805 10.1016/j.anai.2015.05.011 PMC4593731

[7] Shenoy ES , Macy E , Rowe T , Blumenthal KG . Evaluation and management of Penicillin allergy: a review. JAMA 2019;321:188–199. 30644987 10.1001/jama.2018.19283

[8] Patterson RA , Stankewicz HA . Penicillin Allergy. In StatPearls. Treasure Island (FL)2024. 29083777

[9] Lee RU . Penicillin allergy delabeling can decrease antibiotic resistance, reduce costs, and optimize patient outcomes. Fed Pract 2020;37:460–465. 33132684 10.12788/fp.0040 PMC7592897

[10] del Real GA , Rose ME , Ramirez-Atamoros MT , et al. Penicillin skin testing in patients with a history of beta-lactam allergy. Ann Allergy Asthma Immunol 2007;98:355–359. 17458432 10.1016/S1081-1206(10)60882-4

[11] Ham Y , Sukerman ES , Lewis JS, 2nd , Tucker KJ , Yu DL , Joshi SR . Safety and efficacy of direct two-step penicillin challenges with an inpatient pharmacist-driven allergy evaluation. Allergy Asthma Proc 2021;42:153–159. 33685561 10.2500/aap.2021.42.200128 PMC8133016

[12] Holten KB , Onusko EM . Appropriate prescribing of oral beta-lactam antibiotics. Am Fam Physician 2000;62:611–620. 10950216

[13] Freifeld AG , Bow EJ , Sepkowitz KA , et al. Clinical practice guideline for the use of antimicrobial agents in neutropenic patients with cancer: 2010 update by the infectious diseases society of america. Clin Infect Dis 2011;52:e56–93. 21258094 10.1093/cid/cir073

[14] Tamma PD , Avdic E , Li DX , Dzintars K , Cosgrove SE . Association of adverse events with antibiotic use in hospitalized patients. JAMA Intern Med 2017;177:1308–1315. 28604925 10.1001/jamainternmed.2017.1938 PMC5710569

[15] Kaminsky LW , Ghahramani A , Hussein R , Al-Shaikhly T . Penicillin allergy label is associated with worse clinical outcomes in Bacterial Pneumonia. J Allergy Clin Immunol Pract 2022;10:3262–3269. 36182647 10.1016/j.jaip.2022.08.027 PMC10129071

[16] Macy E , Contreras R . Health care use and serious infection prevalence associated with penicillin “allergy” in hospitalized patients: A cohort study. J Allergy Clin Immunol 2014;133:790–796. 24188976 10.1016/j.jaci.2013.09.021

[17] Jeffres MN , Narayanan PP , Shuster JE , Schramm GE . Consequences of avoiding beta-lactams in patients with beta-lactam allergies. J Allergy Clin Immunol 2016;137:1148–1153. 26688516 10.1016/j.jaci.2015.10.026

[18] Charneski L , Deshpande G , Smith SW . Impact of an antimicrobial allergy label in the medical record on clinical outcomes in hospitalized patients. Pharmacotherapy 2011;31:742–747. 21923600 10.1592/phco.31.8.742

[19] Trubiano JA , Leung VK , Chu MY , Worth LJ , Slavin MA , Thursky KA . The impact of antimicrobial allergy labels on antimicrobial usage in cancer patients. Antimicrob Resist Infect Control 2015;4:23. 26034582 10.1186/s13756-015-0063-6 PMC4450507

[20] Blumenthal KG , Lu N , Zhang Y , Li Y , Walensky RP , Choi HK . Risk of meticillin resistant Staphylococcus aureus and Clostridium difficile in patients with a documented penicillin allergy: population based matched cohort study. BMJ 2018;361:k2400. 29950489 10.1136/bmj.k2400 PMC6019853

[21] Cusatis R LC , Feng Z , Allbee-Johnson M , Shaw BE . CIBMTR Patient-Reported Outcomes Data Collection: 2020-2023 PRO Summary Slides. https://cibmtr.org/Files/Summary-Slides--Reports/The-US-Summary-Slides-2022-v4---web-version.pptx. Accessed October 11, 2022.

[22] Harris PA , Taylor R , Minor BL , et al. The REDCap consortium: building an international community of software platform partners. J Biomed Inform 2019;95:103208. 31078660 10.1016/j.jbi.2019.103208 PMC7254481

[23] Harris PA , Taylor R , Thielke R , Payne J , Gonzalez N , Conde JG . Research electronic data capture (REDCap)--a metadata-driven methodology and workflow process for providing translational research informatics support. Journal of Biomed Inform 2009;42:377–381. 10.1016/j.jbi.2008.08.010 PMC2700030 18929686

[24] Al-Hasan MN , Acker EC , Kohn JE , Bookstaver PB , Justo JA . Impact of Penicillin allergy on empirical carbapenem use in gram-negative bloodstream infections: an antimicrobial stewardship opportunity. Pharmacotherapy 2018;38:42–50. 29105102 10.1002/phar.2054

[25] Baxter M , Bethune C , Powell R , Morgan M . Point prevalence of penicillin allergy in hospital inpatients. J Hosp Infect 2020;106:65–70. 32553856 10.1016/j.jhin.2020.06.016

[26] Macy E , Shu YH . The effect of penicillin allergy testing on future health care utilization: a matched cohort study. J Allergy Clin Immunol Pract 2017;5:705–710. 28366717 10.1016/j.jaip.2017.02.012

[27] Li M , Krishna MT , Razaq S , Pillay D . A real-time prospective evaluation of clinical pharmaco-economic impact of diagnostic label of ‘penicillin allergy’ in a UK teaching hospital. J Clin Pathol 2014;67:1088–1092. 25185139 10.1136/jclinpath-2014-202438

[28] Blumenthal KG , Li Y , Banerji A , Yun BJ , Long AA , Walensky RP . The cost of Penicillin allergy evaluation. J Allergy Clin Immunol Pract 2018;6:1019–1027 e1012. 28958738 10.1016/j.jaip.2017.08.006 PMC5862726

