# Supplementary material for: Impact of beta-lactam allergy labels on bone marrow transplant patients
Source: Antimicrob Steward Healthc Epidemiol. 2025 Jun 16;5(1):e130. doi: 10.1017/ash.2025.172 (PMC12171917; doi:10.1017/ash.2025.172)
Supplement: Haxby et al. supplementary material [file S2732494X2500172Xsup001.docx]

**E-Supplement Material**

**eTable 1. Multivariable Regression for Readmission Following Index Encounter**

| **Predictor of Readmission** | **OR** | **95% CI** | **p-value** |
| --- | --- | --- | --- |
| Allergy Label | 1.02 | .054 – 1.91 | 0.955 |
| Transplant Type* |  |  |  |
| Allogeneic Transplant | 6.24 | 2.95 – 9.75 | 0.002 |
| Autologous Transplant | REF | - | - |
| Transplant Indication* |  |  |  |
| Acute Lymphocytic Leukemia | 1.49 | 0.23 – 9.64 | 0.777 |
| Acute Myeloid Leukemia | 1.07 | 0.18 – 6.27 | 0.535 |
| Non-Hodgkin’s Lymphoma | 1.85 | 0.34 – 10.24 | 0.411 |
| Multiple Myeloma | 1.33 | 0.25 – 6.93 | 0.994 |
| Other | 1.36 | 0.22 – 8.62 | 0.942 |
| Hodgkin’s Lymphoma | REF | - | - |

*Independent predictors of readmission within 100 days following BMT. * Denotes confounding variables on the primary exposure variable allergy label.*

**eTable 2. Multivariable Regression Model for ICU Admission**

| **Predictor of ICU Admission** | **OR** | **95% CI** | **p-value** |
| --- | --- | --- | --- |
| Allergy Label | 1.02 | 0.45 - 2.31 | .965 |
| Chronic Kidney Disease* | 5.52 | 2.50 – 12.18 | <.0001 |
| Gram Positive Blood Culture* | 3.47 | 1.49 – 8.05 | .004 |
| Transplant Type* |  |  |  |
| Allogeneic Transplant | 1.54 | 0.33 – 7.16 | 0.580 |
| Autologous Transplant | REF | - | - |
| Transplant Indication* |  |  |  |
| Acute Lymphocytic Leukemia | 5.79 | 0.71 – 47.5 | 0.373 |
| Acute Myeloid Leukemia | 3.47 | 0.50 – 24.13 | 0.990 |
| Hodgkin’s Lymphoma | 5.97 | 1.05 – 33.89 | 0.407 |
| Non-Hodgkin’s Lymphoma | 3.33 | 0.75 – 14.79 | 0.949 |
| Other | 4.22 | 0.59 – 30.03 | 0.711 |
| Multiple Myeloma | REF |  |  |

*Independent predictors of ICU admission within 100 days following BMT. * Denotes confounding variables on the primary exposure variable allergy label.*
